# Restorative functions of Autologous Stem Leydig Cell transplantation in a Testosterone-deficient non-human primate model

**DOI:** 10.7150/thno.46854

**Published:** 2020-07-09

**Authors:** Kai Xia, Hong Chen, Jiancheng Wang, Xin Feng, Yong Gao, Yi Wang, Rongda Deng, Chunxing Wu, Peng Luo, Min Zhang, Chao Wang, Yong Zhang, Yadong Zhang, Guihua Liu, Xiang'an Tu, Xiangzhou Sun, Weiqiang Li, Qiong Ke, Chunhua Deng, Andy Peng Xiang

**Affiliations:** 1 Department of Andrology, The First Affiliated Hospital, Sun Yat-sen University, Guangzhou, Guangdong, China.; 2 Center for Stem Cell Biology and Tissue Engineering, Key Laboratory for Stem Cells and Tissue Engineering, Ministry of Education, Sun Yat-sen University, Guangzhou, Guangdong, China.; 3 Reproductive Medicine Center, The Key Laboratory for Reproductive Medicine of Guangdong Province, The First Affiliated Hospital, Sun Yat-sen University, Guangzhou, Guangdong, China.; 4 Center for Reproductive Medicine, The Sixth Affiliated Hospital, Sun Yat-sen University, Guangzhou, Guangdong, China.; 5 Guangdong Provincial Key Laboratory of Orthopedics and Traumatology, The First Affiliated Hospital, Sun Yat-sen University, Guangzhou, Guangdong, China.; 6 Department of Biochemistry, Zhongshan School of Medicine, Sun Yat-sen University, Guangzhou, Guangdong, China.; 7 Department of Nuclear Medicine, The Third Affiliated Hospital, Sun Yat-sen University, Guangzhou, Guangdong, China.; 8 Scientific Research Center, The Seventh Affiliated Hospital of Sun Yat-sen University, Shenzhen, Guangdong, China.

**Keywords:** Stem Leydig cell, Autologous, Transplantation, Testosterone deficiency, Non-human primate

## Abstract

**Rationale:** Stem Leydig cells (SLCs) transplantation can restore testosterone production in rodent models and is thus a potential solution for treating testosterone deficiency (TD). However, it remains unknown whether these favorable effects will be reproduced in more clinically relevant large-animal models. Therefore, we assessed the feasibility, safety and efficacy of autologous SLCs transplantation in a testosterone-deficient non-human primate (NHP) model.

**Methods:** Cynomolgus monkey SLCs (CM-SLCs) were isolated from testis biopsies of elderly (> 19 years) cynomolgus monkeys by flow cytometry. Autologous CM-SLCs were injected into the testicular interstitium of 7 monkeys. Another 4 monkeys were injected the same way with cynomolgus monkey dermal fibroblasts (CM-DFs) as controls. The animals were then examined for sex hormones, semen, body composition, grip strength, and exercise activity.

**Results:** We first isolated CD271^+^ CM-SLCs which were confirmed to expand continuously and show potential to differentiate into testosterone-producing Leydig cells (LCs) *in vitro*. Compared with CM-DFs transplantation, engraftment of autologous CM-SLCs into elderly monkeys could significantly increase the serum testosterone level in a physiological pattern for 8 weeks, without any need for immunosuppression. Importantly, CM-SLCs transplantation recovered spermatogenesis and ameliorated TD-related symptoms, such as those related to body fat mass, lean mass, bone mineral density, strength and exercise capacity.

**Conclusion:** For the first time, our short-term observations demonstrated that autologous SLCs can increase testosterone levels and ameliorate relevant TD symptoms in primate models. A larger cohort with long-term follow-up will be required to assess the translational potential of autologous SLCs for TD therapy.

## Introduction

Testosterone deficiency (TD) occurs in approximately 20% of men aged 40 to 79 years, and the incidence increases with age 1-4. The clinical features of TD include sexual dysfunction 5, obesity 2, muscle weakness 6, 7, osteoporosis 8, 9 and others. Epidemiological studies have suggested that low testosterone levels are associated with multiple diseases and deteriorated quality of life 10, 11. Exogenous testosterone replacement therapy (TRT) is a straightforward treatment for male TD 4, 10, 12 but may cause a series of adverse effects, such as stroke 13, heart attack 14 and prostate tumorigenesis 15, 16. More importantly, this treatment disrupts the hypothalamic-pituitary-gonadal axis (HPG axis) 17, reducing the intratesticular testosterone concentration and disturbing spermatogenesis 18-20; it therefore fails to meet the requirements of individualized treatment 11, especially given that the physiological testosterone level varies across individuals 21. Thus, we need to explore promising new approaches for testosterone supplementation in a physiological pattern.

Leydig cells (LCs) are the primary source of circulating testosterone. However, their function is impaired in aged individuals, which appears to be due to a reduction in steroidogenic capacity per LC 22. In addition, the total number of LCs was reported to decline over 40% between young (20-48 years old) and aged (50-76 years old) men 23. Collectively, these studies suggested that aging-associated TD is attributed to not only impaired function but also a reduced number of LCs, while hypothalamic-pituitary function remains relatively unaffected 24. In this context, transplantation of LCs seems to be an ideal candidate for physiological and long-acting testosterone delivery 25-27. However, the limited proliferative capacity of adult LCs has restricted their translational application. Stem Leydig cells (SLCs), with the ability to proliferate and differentiate into LCs, could be a potential replacement 28-30. Transplanted rodent SLCs have been shown to replace damaged or senescent LCs and restore testosterone production 31-34. Thus, SLCs are considered to be a favorable cell source for the treatment of TD. Very recently, we showed for the first time that CD271 (p75 neurotrophin receptor)-positive cells in human testes from young donors (18-32 years of age) have the characteristics of SLCs. Importantly, transplanted CD271^+^ SLCs were shown to restore testosterone production in a circadian rhythm and promote the recovery of spermatogenesis in a LC-disrupted model in rats 35. These findings provide new insights into the clinical application of SLCs.

Autologous stem cell transplantation overcomes the current limitations posed by allogeneic donor cells and raises the enticing possibility that adult stem cells could be used to autologously replace diseased or damaged tissue 36-38. It is not yet known whether SLCs can be isolated and propagated from elderly patients with the goal of treating age-related TD with autologous transplantation. Furthermore, it remains unclear whether the favorable testosterone restoration effects seen in small-animal models will be reproduced in more clinically relevant large-animal models. Non-human primates (NHPs) are similar to humans in size, behavior, physiology, biochemistry, structure and organ function 39, 40. Researches on NHPs can directly address relevant and challenging translational aspects of cell transplantation therapy and form a translational bridge from small-animal models to humans 41. Previous studies have demonstrated that NHP models offer unique opportunities to develop various stem cell-based therapeutic interventions, including those based on spermatogonial stem cells (SSCs) 42, pluripotent stem cell-derived cardiomyocytes 43, mesenchymal stem cells 44 and neural stem cells 45. Using a cynomolgus monkey model, Morizane et al. showed that compared with allogeneic grafts, the autologous transplantation of iPSC-derived neural cells is advantageous for minimizing the immune response in the brain 38. Male cynomolgus monkeys (*Macaca fascicularis*) show characteristic age-associated physiological changes comparable to those of human males 46-48, which makes them an ideal animal model for translational research, such as developing appropriate testosterone supplementation paradigms and evaluating the potential of stem cell transplantation in this context 49, 50. Thus, preclinical assessment of the feasibility, safety and efficacy of autologous SLCs transplantation in NHP models of TD will be an important step in determining the translational potential of this cell therapy.

## Methods

### Animals

8-week-old male immunodeficient NCG mice (Nanjing Biomedical Research Institute of Nanjing University) were used for our analysis of tumorigenicity. The mice were maintained under controlled temperature (24 ± 1°C) and relative humidity (50-60%), with a 12-h light/12-h dark cycle and free access to a standard rodent diet and drinking water. Male cynomolgus monkeys (*Macaca fascicularis*; Blooming Spring Biological Technology Development Co., Ltd.) were used for experiments. The details are specified in Table S1. All monkeys were housed at the Blooming Spring Biological Technology Development Co., Ltd., which is fully accredited by the Association for Assessment and Accreditation of Laboratory Animal Care (AAALAC). All animal experiments were carried out using protocols approved by the Ethics Committee of Zhongshan School of Medicine, Sun Yat-sen University (No. 2018-202), and the ICE for Clinical Research and Animal Trials of the First Affiliated Hospital of Sun Yat-sen University (No. 2019-013).

### Isolation and culture of CM-SLCs from donor monkey testes

Testis tissue samples were collected from cynomolgus monkeys by subcapsular biopsy 51. Less than 1% of the testicular parenchyma was removed (0.4-0.6 g) with puncturing forceps, which were applied to the lateral side of the testis. SLCs were isolated as previously reported 32. In detail, the interstitial cells were dissociated from the seminiferous tubules by digestion with 1 mg/mL collagenase type IV (Gibco, Grand Island, NY, USA) in Dulbecco's modified Eagle's medium/Nutrient Mixture F-12 (DMEM/F12; 1:1, Gibco, Grand Island, NY, USA) at 37°C for 15 min. DMEM/F12 containing 5% fetal bovine serum (FBS, HyClone, Logan, UT, USA) was added to stop the digestion, and each sample was filtered through a 45 μm filter. The samples were centrifuged at 250 g at 4°C for 4 min, and the pellet was washed twice with phosphate-buffered saline (PBS) and then incubated with AF647-conjugated anti-CD271 (BD Biosciences, Franklin Lakes, NJ, USA) and isotype antibody (BD Bioscience) in the dark for 15 min. The CD271^+^ cells were enriched by flow cytometry using an Influx Cell Sorter (BD Bioscience). The expansion medium was a modified version of the previously published recipe 35 and consisted of DMEM/F12 (Gibco) supplemented with 1% nonessential amino acids (NEAA, Gibco), insulin-transferrin-sodium selenite (ITS, Gibco), N2 (Gibco), 2% B27 (Gibco), 20 ng/mL basic fibroblast growth factor (bFGF, PeproTech, Rocky Hill, NJ, USA), epidermal growth factor (EGF, PeproTech), platelet-derived growth factor-BB (PDGF-BB, PeproTech), oncostatin M (OSM, PeproTech), 1 ng/mL leukemia inhibitory factor (LIF, Millipore, Bedford, MA, USA), 1 nM dexamethasone (Sigma-Aldrich, St. Louis, MO, USA), and 0.1 mM β-mercaptoethanol (Gibco). The cultures were kept at 37°C in a humidified 5% CO_2_ water-jacketed incubator (Thermo Fisher, Marietta, OH, USA), and the medium was changed every 3 days.

### Cell proliferation assay

Cell proliferation was assessed using our previously reported method 35. Cells at different passages (P5, P10, P15, and P20) were seeded into 24‐well plates at a density of 1×10^4^ cells/well. Cell proliferation was examined at set times using a Cellometer Auto T4 automated cell counter (Nexcelom Bioscience, Lawrence, MA, USA).

### Colony formation assay

Colony formation was assessed using our previously reported method 32. CM-SLCs were dissociated into a single-cell suspension and diluted to a density of 500 cells/mL, and 2 µL of the diluted cell suspension and 150 µL of expansion medium were plated in each well of a 96-well plate (Corning, Tewksbury, MA, USA). Wells containing only one cell were marked and observed daily. Spheres were defined as free-floating spherical structures with a diameter > 50 μm.

### Multilineage differentiation of CM-SLCs *in vitro*

For osteogenic, adipogenic and chondrogenic differentiation, CD271^+^ cells were cultured in differentiation-inducing medium for 2-4 weeks and analyzed by staining with Alizarin Red (Sigma-Aldrich), Oil Red O (Sigma-Aldrich) and Toluidine Blue (Sigma-Aldrich), respectively, as previously described 52. The expression levels of lineage-specific genes were analyzed by RT-PCR, including alkaline phosphatase (ALP), secreted protein acidic and cysteine rich (SPARC) and runt-related transcription factor 2 (RUNX2) for osteogenesis, fatty acid-binding protein 4 (FABP4) and peroxisome proliferator activated receptor γ (PPARγ), and collagen type 2 alpha 1 chain (COL2A1) and aggrecan (ACAN) for chondrogenesis (primers are listed in Table S4).

### LCs differentiation of CM-SLCs* in vitro*

For LCs lineage differentiation, the SLCs were plated in fresh differentiation-inducing medium containing phenol red-free DMEM/F12 (Gibco), 2% FBS (HyClone), 1 ng/mL luteinizing hormone (LH, R&D, Minneapolis, MN, USA), 1 nM thyroid hormone (Sigma-Aldrich), 10 ng/mL platelet-derived growth factor-AA (PDGF-AA, PeproTech), 50 ng/mL insulin-like growth factor 1 (IGF1, PeproTech), and 1% ITS (Gibco) and incubated for up to 28 days as previously described 52. Cell culture supernatants were collected for testosterone detection at the indicated time points. Differentiation was confirmed by immunostaining and RT-PCR for LCs lineage markers (primers and antibodies are listed in Table S4-5).

### Identification of TD cynomolgus monkeys

To screen for the cynomolgus monkey TD model, 30 elderly monkeys (19 to 23 years old) were initially included 40. Blood samples were collected at 9-10 a.m. three times a week, and the levels of total testosterone (TT) and free testosterone (FT) were measured 53. Based on the obtained results, 11 monkeys (TT < 10 ng/mL and FT < 0.25 ng/mL) were enrolled in the study. The animals were randomly assigned to two groups: group 1 received CM-DFs transplantation (M1-M4, n=4), while group 2 received CM-SLCs transplantation (M5-M11, n=7) (a more detailed description is provided in Table S1).

### Lentiviral treatment of transplanted cells

For autologous transplantation, CM-SLCs or CM-DFs were treated with lentiviruses encoding red fluorescent protein (RFP). The lentiviral expression vector was designated pLent-CAG-mCherry-P2A-Puro (Vigene Biosciences, Rockville, MD, USA).

### Cell transplantation

The cell transplantation procedure was modified from the previously described method 42. Cells were digested and suspended at approximately 3×10^7^ cells/mL in PBS containing a 20% concentration of the microbubble ultrasound contrast agent SonoVue (Bracco Imaging, HW, UK) in a total volume of 500 µL. Cell transplantation was performed using ultrasound-guided testicular injections (Movie S1). For this purpose, a 13-MHz linear superficial probe was used to visualize the testis on an ultrasound machine Logiq E9 (GE, Boston, MA, USA) and guide a 25G spinal needle into the testis.

### Immunofluorescence staining

For immunofluorescence staining of cultured cells, the cells were fixed with 4% paraformaldehyde (PFA, Phygene, Fujian, China) for 15 min, permeabilized with 0.2% Triton X-100 (Sigma-Aldrich) for 15 min and blocked with 3% bovine serum albumin (BSA, Sigma-Aldrich) for 30 min at room temperature, as described previously 32. These cells were incubated overnight with the relevant primary antibodies at 4-8°C. Then, the cells were washed with PBS and then incubated with the appropriate secondary antibodies for 45 min at room temperature. Nuclei were counterstained with DAPI (Gibco) for 5 min at room temperature. For immunofluorescence staining of monkey testes, the tissues were fixed in 4% PFA (Phygene), dehydrated with 30% sucrose (Sangon Biotech, Shanghai, China), and sectioned at a thickness of 10-50 μm. The sections were permeabilized for 30 min using 0.2% Triton X-100 (Sigma-Aldrich), blocked with 3% BSA (Sigma-Aldrich) in PBS for 45 min at room temperature and then incubated overnight with the relevant primary antibodies. The sections were then washed with PBS, incubated with the appropriate secondary antibodies for 45 min at room temperature, and co-stained with DAPI for 5 min (antibodies are listed in Table S5). Images were captured with an LSM800 confocal microscope (Zeiss, Jena, Germany) and a Dragonfly CR-DFLY-202 2540 (Andor Technology, Belfast, UK).

### RT-PCR analysis

The total RNA fraction was extracted using a RNeasy Mini kit (Qiagen, Germantown, MD, USA) and reverse-transcribed using a High-Capacity RNA-to-cDNA^TM^ Kit (Thermo Fisher). The obtained mRNA (1 μg) was incubated with 1× RT Buffer Mix and 1× RT Enzyme Mix. The reaction was incubated at 37°C for 60 min to generate cDNA, and then the reaction was stopped by heating to 95°C for 5 min and held at 4°C. Standard PCR experiments were performed using Taq DNA polymerase (NEB, Ipswich, MA, USA), and the relevant primers are listed in Table S4.

### Karyotype analysis

G-band chromosomal analysis was performed by DAAN Gene Co., Ltd.

### Tumorigenesis assay

Tumorigenesis was assessed as previously reported 54. Expanded CM-SLCs or MA-10 Leydig tumor cells were suspended in 200 μL of PBS and injected subcutaneously into immunodeficient NCG mice (Nanjing Biomedical Research Institute of Nanjing University). Tissues were harvested for analysis at the time of tumor detection or at 3 months post injection if no detectable tumor formed.

### Safety evaluation

Total testosterone levels, high-sensitivity C-reactive protein (Hs-CRP), hematological parameters (white blood cell, neutrophil, lymphocyte, monocyte, eosinophil, basophil, red blood cell, hemoglobin and platelet counts) were measured at baseline and at weeks 2 and 4 post testicular biopsy. Hematological parameters (white blood cell, neutrophil, lymphocyte, monocyte, eosinophil, basophil, red blood cell, hemoglobin and platelet counts), liver function (aspartate aminotransferase, alanine aminotransferase, alkaline phosphatase, cholinesterase, total protein, globulin, albumin, prealbumin, total bilirubin, direct bilirubin, indirect bilirubin, total bile acid, lactate dehydrogenase and γ-glutamyl transferase levels), kidney function (urea, uric acid and creatinine levels), heart function (creatinine kinase, creatinine kinase isoenzyme and hydroxybutyrate dehydrogenase levels) and tumor markers (alpha-fetoprotein, β-human chorionic gonadotropin and prostate-specific antigen) were measured at baseline and at weeks 4 and 8 post transplantation. All laboratory measurements were performed with standard autoanalyzer methods at the First Affiliated Hospital of Sun Yat-sen University.

### Sex hormone concentration assay

To evaluate testosterone production after cell transplantation, blood samples were collected from the upper limb veins at weeks 0, 1, 2, 3, 4, 6, 8, 10 and 12 after cell transplantation. To test whether the testosterone level exhibited a circadian rhythm after CM-SLCs transplantation, M6 and M8 were chosen randomly to represent the group. Eight weeks after transplantation, blood samples were taken over a 36‐h period at 08:00, 14:00, and 20:00 and 02:00, 08:00, 14:00, and 20:00 the next day. To test whether testosterone secretion was modulated by the HPG axis 17, M6 and M8 were subcutaneously administered decapeptyl (5 μg/kg/day) (Ipsen Pharma, Boulogne-Billancourt, France) for 28 consecutive days from 8 weeks after CM-SLCs transplantation. Blood samples were collected on days 0, 1, 4, 7, 10, 15, 20 and 25 post injection. Centrifugation was performed to obtain serum samples (3000 g, 10 min), which were stored at -80°C until analysis 55. ELISA kits were used to analyze serum testosterone (R&D) and luteinizing hormone (Cusabio, Wuhan, Hubei, China). Absorbance at 450 nm was determined using an ELISA microtiter plate reader (Tecan, TECAN, SunriseTM, Männedorf, Switzerland). The hormone concentrations were calculated by reference to standard curves, which were constructed by graphing the absorbance of each reference standard against its corresponding concentration.

### Semen collection and analysis

Semen samples from experimental animals were analyzed at weekly intervals before and after cell transplantation as previously described 56. The samples were collected into sterile collection tubes and allowed to liquefy at 37°C for 30 min before evaluation. The volume was recorded, and the liquid fraction was resuspended in 90 μL of 37°C Tyrode albumin lactate pyruvate (TALP)-HEPES with BSA (Sigma-Aldrich) and centrifuged at 130-150 g for 10 min. The sample was washed and resuspended, and then the sperm count and concentration were measured on a hemocytometer. The percentage of motility was determined from duplicate counts of 100 sperm performed using a phase-contrast microscope (Leica, Wetzlar, Germany).

### Body composition assessment

Dual-energy X-ray absorptiometry machine Discovery A (Hologic, Bedford, MA, USA) was used to assess total body fat, lean tissue mass and bone mineral density, as previously described 5, 9, 57. Measurements were taken at baseline and at weeks 8-10 of the study. This technique yields data for fat mass, lean mass and bone mineral density for the total body and definable regions of interest. For scans, anesthesia was induced and maintained by intramuscular injection of ketamine (2 mg/kg) (CAHG, Beijing, China).

### Grip strength evaluation

Grip strength was measured using a method adapted from those previously described for rodents and marmosets 58. For each evaluation, a monkey entered the cage (2 feet long × 2 feet wide × 3 feet high), and grip strength was assessed during retrieval of food objects placed in a cup in front of the cage. Grip strength was recorded with a digital push-pull gauge (Handpi, Leqing, Zhejiang, China) connected to the food cup. The procedure was repeated at least five times per individual, and the maximum force was recorded for each grip attempt with both hands. The highest measurement within the testing session was considered to be the maximum grip strength of the animal at that time.

### Exercise activity assessment

Exercise activity was evaluated weekly throughout the study using an open-field task modified from those previously described by other groups 45. For each assessment, a monkey entered the open-field-testing enclosure (2 feet long × 2 feet wide × 7 feet high), and climbing was assessed during retrieval of food objects placed in a cup hanging at a 6 feet height in front of the cage. Assessments in this open field were performed with two observers: one videotaped the session, and one recorded the climbing distance and time on a scoring sheet. The videos were used to confirm the live scoring data. All assessments were performed without knowledge of group inclusion.

### Statistical analysis

All results represent data from at least three independent experiments and are expressed as the mean ± sem. Statistical analyses were performed using GraphPad Prism v6.0c (GraphPad software). All statistical comparisons were made using Student's *t*-test or one-way ANOVA. Differences of P < 0.05 were considered significant.

## Results

### Isolation, characterization and long-term culture of CD271^+^ cells from testis biopsies of elderly cynomolgus monkeys

The isolation of SLCs from primary tissue has been hampered by the limited selectivity of the available markers 29, 59. We previously demonstrated that CD271 (p75 neurotrophin receptor) can be used as a cell surface marker for identifying and isolating SLCs from human testes 35. To further verify whether it could be used as a putative marker for isolating SLCs from NHPs, we first examined the CD271 expression pattern in the testes of cynomolgus monkeys. Consistent with our previous findings, CD271^+^ cells were located in the interstitium of the testis, co-expressed the known SLCs marker Nestin, and showed negligible co-expression of the mature LCs lineage markers 3-beta-hydroxysteroid dehydrogenase (3β-HSD) and steroidogenic acute regulatory protein (StAR) (Figure S1A). Moreover, we observed that CD271 expression gradually decreased with age until the signal was barely detectable in the testicular interstitium of aged monkeys (19 to 23 years old) (Figure S1B).

**Figure 1:**
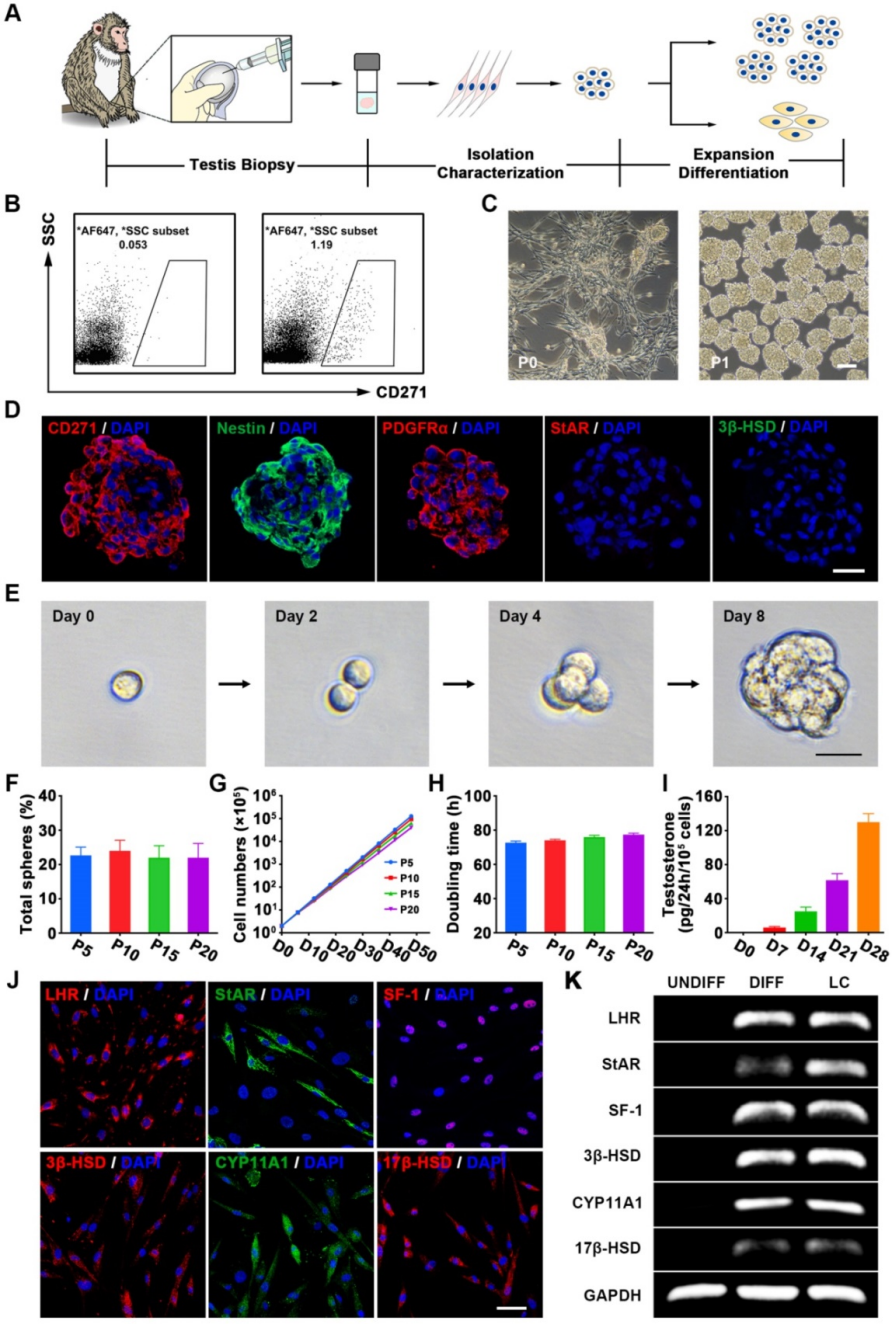
** Characterization of CM-SLCs from the testes of cynomolgus monkeys with TD.** (**A**) Schematic of the experimental procedure used to identify CM-SLCs. (**B**) Flow cytometry was used to isolate CD271^+^ cells from the testes of cynomolgus monkeys with TD. Left: isotype controls. Right: stained samples. SSC: side-scattered light. (**C**) Phase-contrast micrographs of CM-SLCs in primary culture (P0) and passage 1 (P1). Scale bar, 100 µm. (**D**) Cultured CM-SLCs spheres express CD271, Nestin and PDGFRα but not StAR or 3β-HSD. Nuclei were counterstained with DAPI (blue). Scale bar, 25 µm. (**E**) Representative images showing clonal sphere growth from single cells, as observed using a bright-field microscope. Scale bar, 25 µm. (**F**) The frequency of sphere formation from single cells was equivalent at different passages (P5, P10, P15, and P20; n=3). (**G**) The proliferation rates of the isolated CM-SLCs at different passages (P5, P10, P15, and P20) were similar (n=3). (**H**) The average population-doubling times of cells at different passages (P5, P10, P15, and P20) were similar (n=3). (**I**) Testosterone production progressively increased during culture of CD271^+^ cells in differentiation-inducing medium (DIM; n=3). (**J**) At 14 days after differentiation, immunofluorescence staining showed that the differentiated cells clearly expressed the LCs lineage-specific markers LHR, StAR, SF-1, 3β-HSD, CYP11A1 and 17β-HSD. Scale bar, 50 µm. (**K**) RT-PCR analysis confirmed that the expression of the LCs lineage-specific markers was higher in differentiated cells (DIFF) compared to undifferentiated controls (UNDIFF), in which the markers were undetectable. Data are expressed as the mean ± sem and were analyzed by one-way ANOVA.

To investigate whether SLCs could be isolated from elderly monkey testes using CD271 as a putative surface marker, we obtained testicular biopsies 60 (less than 1% testis tissue) (Table S1) from 11 elderly male cynomolgus monkeys (Figure 1A). There were no significant abnormalities in TT, Hs-CRP or hematological parameters at weeks 2 and 4 after testicular biopsy (Table S2). CD271^+^ cells were isolated by flow cytometry (Figure 1B) and seeded in expansion medium. After 1 day of culture, most cells adhered to the plastic wells. When the adherent cells had propagated to 70-80% confluence, we dissociated these cells and transferred them to a new plate for further expansion. The CD271^+^ cells formed small spheres, which subsequently became floating spheres and showed proliferative ability (Figure 1C and S3A). As control cells, we also isolated dermal fibroblasts (DFs) from each member of the cohort.

Immunofluorescence staining showed that the cytospheres of cultured CD271^+^ cells expressed Nestin and PDGFRα but not the LCs lineage markers StAR and 3β-HSD (Figure 1D) at passage 5 (P5). Cultured CD271^+^ cells of different passages (P5, P10, P15, and P20) did not show any significant difference in clonogenic efficiency (Figure 1E-F), proliferation rate (Figure 1G) or population-doubling time (Figure 1H), indicating that these cells were highly clonogenic and exhibited self-renewal capacity *in vitro*. More importantly, we did not observe any obvious chromosomal elimination, displacement or imbalance after long-term culture (Figure S3C). When the CD271^+^ cells of P5 or P20 were inoculated subcutaneously into 8-week-old immunodeficient NCG mice, we failed to observe any sign of tumorigenicity 3 months later; in contrast, control mice injected with MA-10 Leydig tumor cells exhibited efficient formation of tumors (100%) (Figure S3D).

To investigate their potential to differentiate into LCs *in vitro*, CD271^+^ cells were cultured in differentiation medium. The differentiated cells expressed LCs-related steroidogenic enzymes, including StAR, 3β-HSD, cytochrome p450 family 11 subfamily A member 1 (CYP11A1), 17-beta-hydroxysteroid dehydrogenase (17β-HSD), luteinizing hormone receptor (LHR) and the nuclear transcription factor steroidogenic factor 1 (SF-1), as determined by immunofluorescence staining (Figure 1J and S2) and RT-PCR analysis (Figure 1K). Examination of cell culture supernatants collected at different time points revealed that testosterone synthesis gradually increased over time following the LCs differentiation of CD271^+^ cells (Figure 1I). After long-term culture (P20), the CD271^+^ cells retained their potential to differentiate into LCs (Figure S3B). Furthermore, these cells could differentiate into osteogenic, adipogenic and chondrogenic lineages (Figure S4). Due to their self-renewal and multiple differentiation potential, we designated these CD271^+^ cells as cynomolgus monkey SLCs (CM-SLCs).

### Autologous transplantation of CM-SLCs partially recovers testosterone production

**Figure 2:**
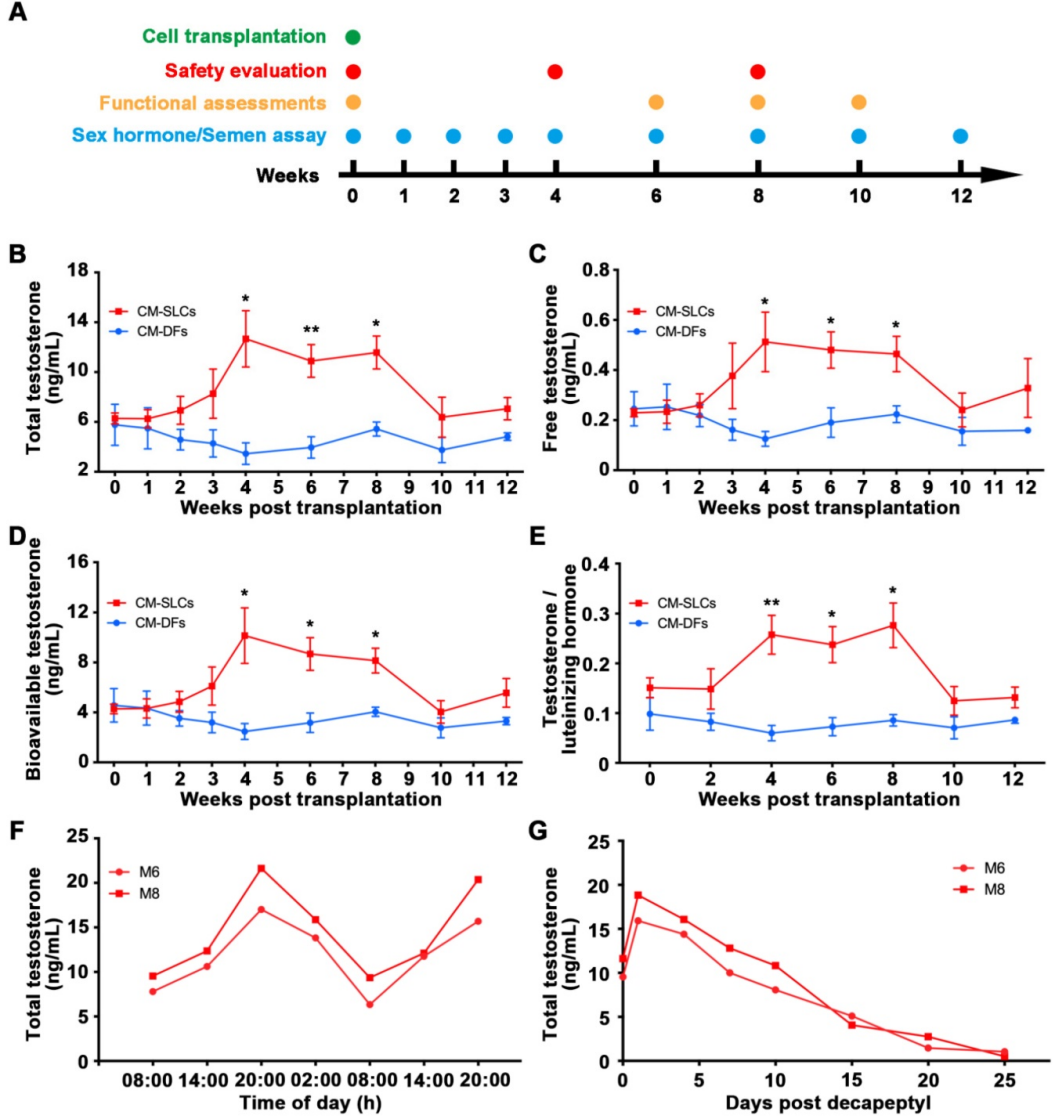
** CM-SLCs transplantation recovers testosterone levels in monkeys with TD.** (**A**) Schematic of the experimental procedure used for cell transplantation. (B-D) The concentrations of total testosterone (**B**), free testosterone (**C**), and bioavailable testosterone (**D**) were detected at the indicated time points in both groups. (**E**) Total testosterone/luteinizing hormone was measured at the indicated time points in both groups. (**F**) The cynomolgus monkeys (M6 and M8) in the CM-SLCs transplanted group appeared to exhibit a circadian rhythm of testosterone secretion. (**G**) The testosterone secretion ability of CM-SLCs monkeys (M6 and M8) was suppressed by decapeptyl treatment. Data are expressed as the mean ± sem and were analyzed by Student's *t*-test; * P < 0.05, ** P < 0.01.

Aging is generally associated with a progressive decline in the biosynthesis of testosterone by LCs 25, 61. Here, we performed autologous transplantation experiments to investigate whether CM-SLCs could differentiate into LCs *in vivo* and increase testosterone levels in a TD monkey model (Figure 2A). We screened 30 elderly monkeys three times for the TT and FT (see Methods) and then enrolled 11 monkeys that were 19 to 23 years old and had conditions indicative of TD (TT < 10 ng/mL and FT < 0.25 ng/mL). The animals were randomly divided into two groups: group 1 received CM-DFs transplantation (n = 4), while group 2 received CM-SLCs transplantation (n = 7) (Table S1). Cells were injected into the interstitium of the testes by ultrasound-guided testicular injection (Movie S1). Using this approach, we introduced 500 μL of cell suspension into per testis of each recipient. The cell numbers injected per testis ranged from 11.5 to 21.3 × 10^6^ cells (Table S1). We failed to observe any significant acute or chronic adverse effects at weeks 4 and 8 after transplantation of autologous CM-SLCs (Table S3). No animal in this study received any immunosuppressive treatments during the duration of the study.

Blood samples were collected at the indicated time points after transplantation (Figure 2A). Our analysis revealed that the serum concentrations of TT significantly increased from 6.28 ± 0.44 ng/mL before transplantation to 12.67 ± 2.26 ng/mL at week 4 and slowly decreased beyond 8 weeks post transplantation of CM-SLCs (Figure 2B). More importantly, the serum concentrations of FT (Figure 2C) and bioavailable testosterone (BT) (Figure 2D), which is currently considered to be the gold standard for the diagnosis of TD in elderly men 2, 62, were also increased compared with those observed in the CM-DFs group. Moreover, the ratio of TT to luteinizing hormone (LH), which is a marker for LCs function 63, was increased significantly at weeks 4-8 after CM-SLCs transplantation (Figure 2E). 8 weeks after CM-SLCs transplantation, LH decreased significantly (Figure S5).

The circulating concentration of testosterone is regulated by the HPG axis 55, 64. Thus, the ideal treatment for TD should provide physiological testosterone levels, exhibit the appropriate circadian rhythm and be modulated by the HPG axis 35, 52. In the present study, two randomly selected cynomolgus monkeys transplanted with autologous CM-SLCs (M6 and M8) appeared to display a normal daily rhythm of testosterone secretion (Figure 2F). When these subjects were administered decapeptyl 52, an agonist of gonadotropin-releasing hormone (GnRHa), the testosterone concentrations peaked at day 1 and dramatically decreased thereafter (Figure 2G), suggesting that the transplanted CM-SLCs could be regulated by the HPG axis 52. Taken together, these results demonstrate that autologous CM-SLCs transplantation could partially recover testosterone production with physiological properties.

### Transplanted CM-SLCs colonize the interstitium and differentiate to LCs *in vivo*

**Figure 3:**
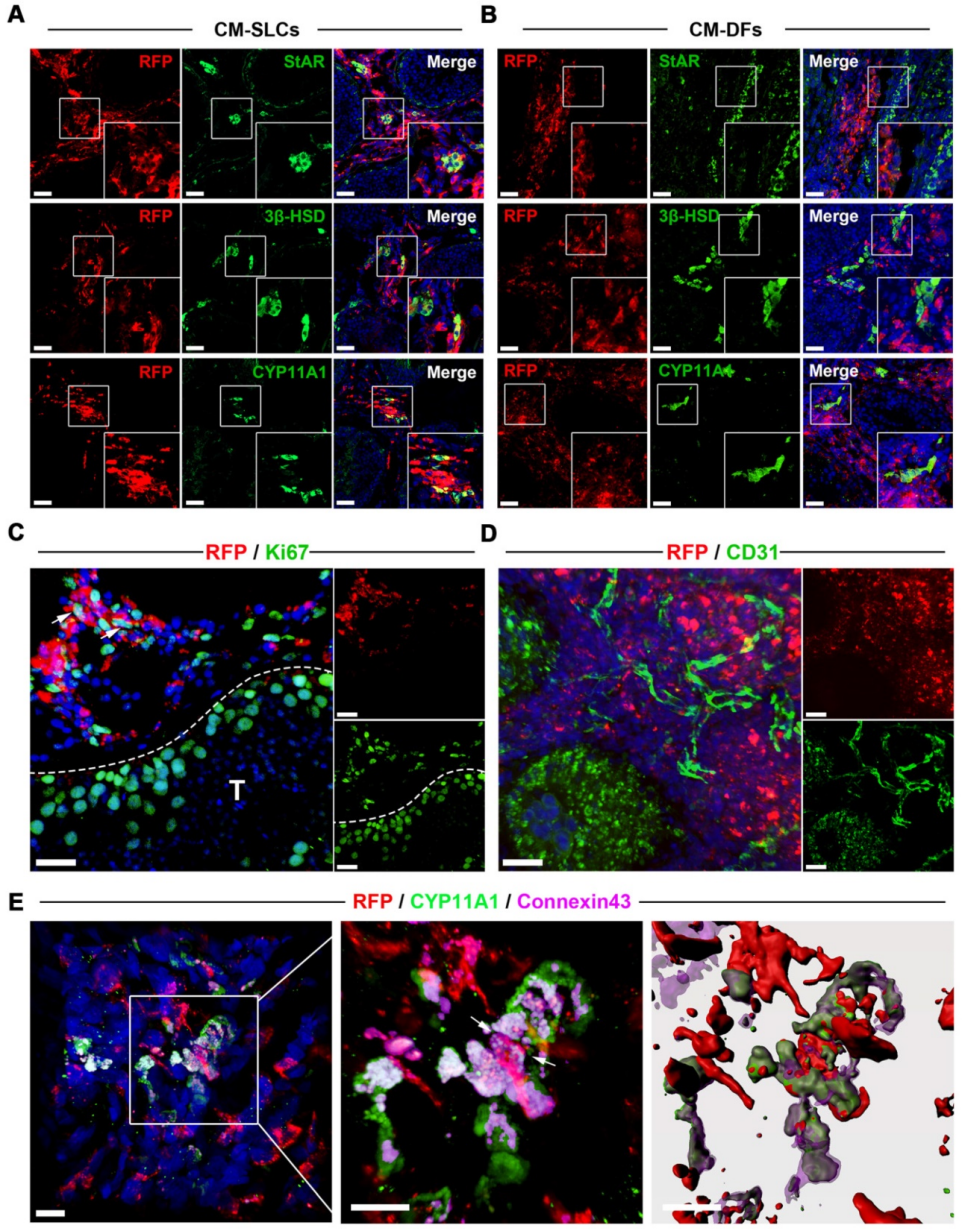
** Transplanted CM-SLCs regenerate Leydig cells in the testes of monkeys with TD.** (**A, B**) Immunostaining shows the accumulation of RFP^+^ CM-SLCs (A), CM-DFs (B) and StAR, 3β-HSD or CYP11A1 in the testicular interstitium of aged monkeys, as assessed at week 8 after transplantation. Scale bar, 40 µm. (**C**) Proliferation of transplanted CM-SLCs was demonstrated by staining for Ki67 at week 8 after transplantation (positive cells are indicated by arrow). T=Seminiferous tubule; Scale bar, 30 µm. (**D**) Vascularization of the transplanted CM-SLCs by host microvessels, as demonstrated by staining of endothelial cells for CD31. Scale bar, 30 µm. (**E**) Expression of the gap junction protein Connexin 43 (Cx43) between the transplanted CM-SLCs and host LCs (indicated by arrow). Nuclei were counterstained with DAPI (blue); Scale bar, 10 µm.

To investigate the* in vivo* fate of the transplanted CM-SLCs, we collected unilateral testis samples from one randomly chosen member of each group (M2 and M5) at weeks 8 and 12 after transplantation, respectively. To distinguish the transplanted cells and their progeny from endogenous cells, we transduced the donor cells with a lentiviral vector expressing mCherry (RFP) driven by the CAG promoter and found that RFP expression did not alter the characteristics of the transplanted cells (data not shown). Immunofluorescence staining showed that the RFP^+^ cells in the CM-SLCs transplantation group were localized exclusively within the interstitium of the testis sample and expressed LCs-specific markers, including StAR (20.12 ± 1.36%), 3β‐HSD (23.03 ± 0.88%) and CYP11A1 (22.15 ± 1.57%) (Figure 3A and S7). These results indicate that some of the transplanted cells have shown the characteristics of LCs. In the CM-DFs group, RFP^+^ cells were found to colonize the interstitium, but no cells expressed any of the LCs markers (Figure 3B). In addition, 9.17 ± 1.98% of the RFP^+^ cells in the CM-SLCs group were positive for the proliferation marker Ki67 (Figure 3C and S7).

Long-term survival of transplanted cells relies on the formation of connections between the graft and the host vascular system 43, 65. Here, we observed that the transplanted cells were connected to host vessels, as evidenced by anti-CD31, von Willebrand Factor (vWF), and VEGF Receptor 2 (VEGFR2) immunostaining without RFP co-expression (Figure 3D and S8). In addition, cell adhesion formation is associated with transplanted cell survival 66, 67. As shown in Figure 3E, the transplanted RFP^+^ cells connected with endogenous LCs through the gap junction protein Connexin 43. These results suggest that CM-SLCs could engraft, survive and integrate into the host microenvironment in the aging testis of TD monkeys.

### CM-SLCs transplantation promotes spermatogenesis

**Figure 4:**
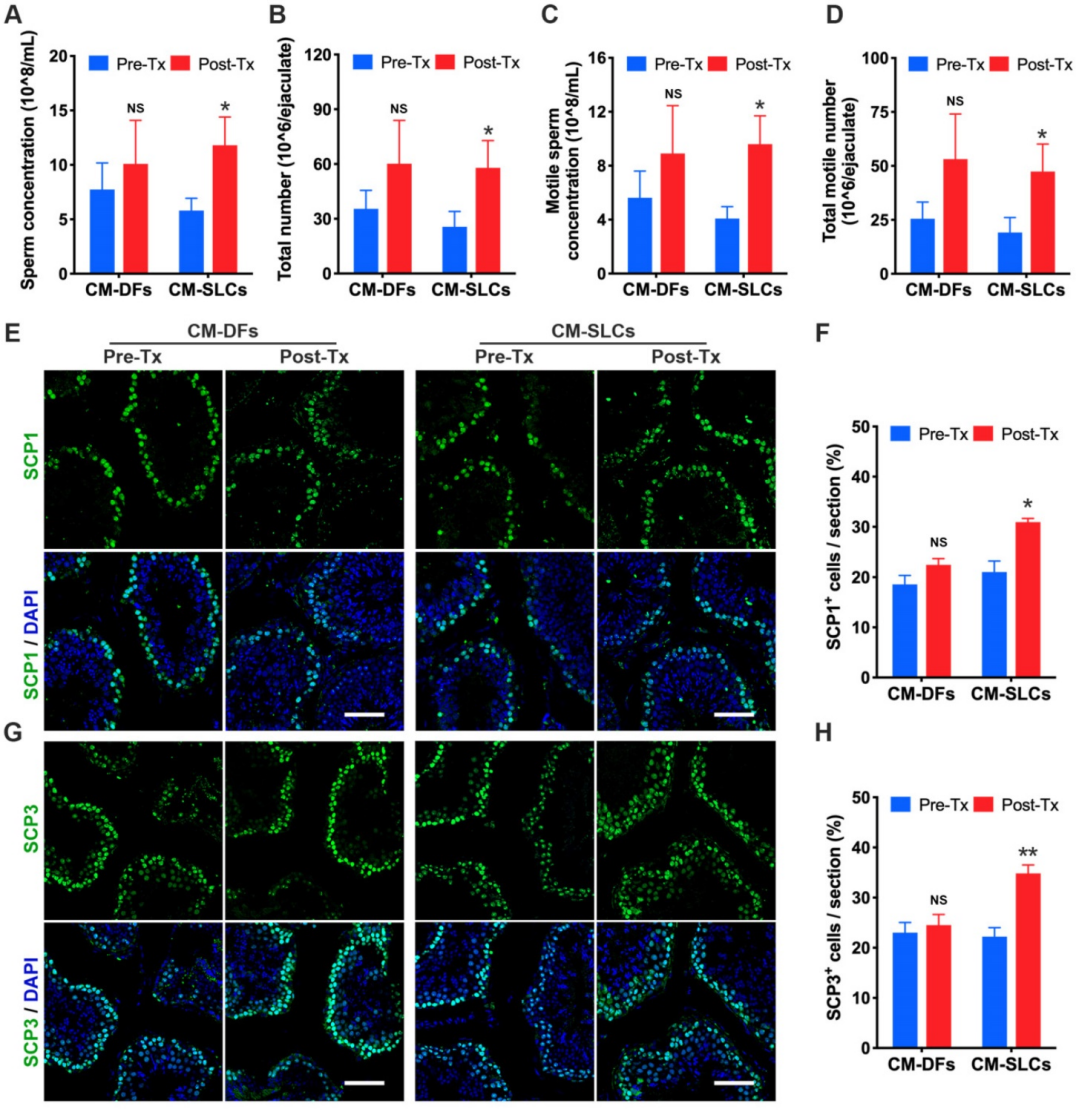
** CM-SLCs transplantation promotes spermatogenesis.** (**A-D**) The sperm concentration (A), total sperm number (B), motile sperm concentration (C), and total motile sperm number (D) were analyzed before and after cell transplantation (n=4 in the CM-DFs group, n=7 in the CM-SLCs group). (**E, G**) Meiotic spermatocytes were observed by immunofluorescence staining for SCP1 (E) and SCP3 (G) antibodies. Scale bar, 75 µm. (**F, H**) Quantitative analysis showing the percentage of SCP1 (F) or SCP3-positive cells (H) in the seminiferous tubules of the testis. Three sections per slide and three slides per testis were counted. Pre-Tx=before transplantation, Post-Tx=after transplantation. Data are expressed as the mean ± sem and were analyzed by Student's *t*-test. * P < 0.05, ** P < 0.01, NS=not significant.

Intratesticular testosterone is essential for spermatogenesis 68, 69, and spermatogenic dysfunction is associated with the aging of LCs 63, 70. To evaluate the impact of CM-SLCs transplantation on spermatogenesis, we performed semen analysis. The CM-SLCs group showed a significant increase in the sperm concentration (Figure 4A), total sperm count (Figure 4B), motile sperm concentration (Figure 4C) and total motile sperm count (Figure 4D) within 6-8 weeks post transplantation (n=7). In contrast, none of these sperm parameters showed a significant change from baseline in the CM-DFs group (n=4; Figure 4A-D).

Since testosterone is also known to be critical for the completion of meiosis 71, we used immunofluorescence staining to examine the expression of the meiosis markers SCP1 and SCP3. Our results showed that SCP1^+^ and SCP3^+^ cells were significantly increased in the testis after transplantation of CM-SLCs but not CM-DFs (Figure 4E-H). Overall, these results demonstrated that the autologous transplantation of CM-SLCs could partially improve spermatogenesis in TD monkeys.

### CM-SLCs transplantation ameliorates TD-related symptoms

**Figure 5:**
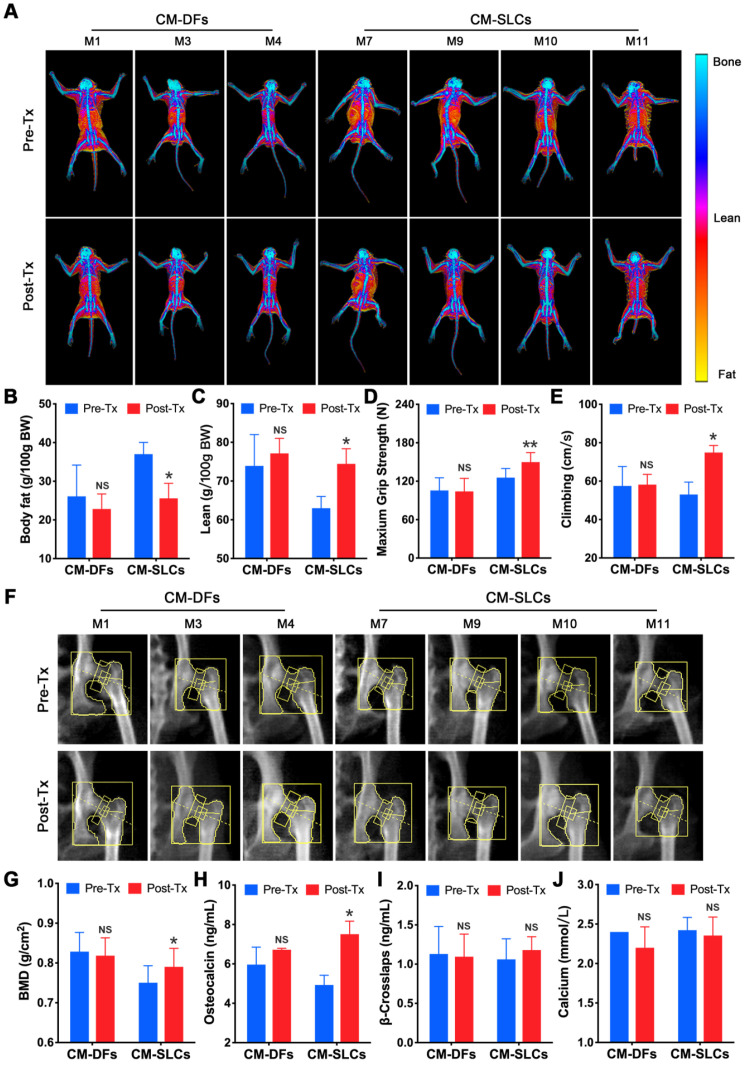
** CM-SLCs transplantation relieves TD-related symptoms.** (**A**) Dual-energy X-ray absorptiometry (DXA) whole-body imaging was performed. (**B, C**) Body fat (B) and lean body percentage (C) were calculated based on the DXA results. (**D**) The maximum grip strength of the two groups was analyzed before transplantation (0 week) and at week 6 after treatment. (**E**) The maximum climbing speeds of the both groups were analyzed before and after transplantation. (**F**) DXA images of the left hip during the study. (**G**) Total bone mineral density (BMD) of the left hip was calculated based on the DXA images. (**H-J**) The serum concentrations of osteocalcin (H), β-crosslaps (I) and calcium (J) were measured before and after cell transplantation (n=3 in the CM-DFs group, n=4 in the CM-SLCs group). Pre-Tx=before transplantation, Post-Tx=after transplantation. Data are expressed as the mean ± sem and were analyzed by Student's *t*-test. * P < 0.05, ** P < 0.01, NS=not significant.

A decrease in testosterone is associated with many symptoms, including obesity 72 and decreases in muscle mass, strength, physical function, bone mass and sexual function 3. To assess the effects of cell transplantation, we detected testosterone deficiency-related symptoms during the study. There was no significant change in body weight for the CM-DFs group (n=3), but this parameter decreased in the CM-SLCs group (n=4) (Figure S9A). Using dual-energy X-ray absorptiometry (DXA), we investigated whether CM-SLCs transplantation resulted in changes in fat and/or lean mass (Figure 5A). Indeed, we found that the total body fat mass and the fat percentage decreased significantly in the CM-SLCs group (n=4), whereas no treatment-related change was detected in the CM-DFs group (n=3) (Figure 5B and S9B). The lean body mass was unchanged in both groups, but the lean percentage significantly increased in the CM-SLCs group (n=4) (Figure 5C and S9C). As previously reported, TD was associated with marked decreases in muscle strength and exercise capacity 73. After transplantation, the maximum grip strength increased starting at week 6 post transplantation in the CM-SLCs group (Figure 5D). In contrast, little change in this parameter was seen among CM-DFs-injected TD animals (Figure 5D). Additionally, exercise capacity notably increased by 8 weeks after CM-SLCs transplantation, but no such increase was detected in the CM-DFs group (Figure 5E).

Previous studies linked TD to reduced bone mineral density (BMD), which can be improved by testosterone replacement treatment 8, 9. Using DXA (Figure 5F), we observed that CM-SLCs transplantation was associated with a mean 6.05% increase in BMD in the total hip 8-10 weeks after transplantation, whereas the same parameter decreased by 0.79% in the CM-DFs group (Figure 5G). Our analysis of bone parameters showed that the mean serum osteocalcin level increased in the CM-SLCs group (n=4), with this difference reaching statistical significance at week 8 post transplantation (Figure 5H). In contrast, no such change was observed in the CM-DFs group (n=3). Neither group showed any transplantation-related difference in the serum concentrations of calcium or β-crosslaps (Figure 5I-J). Taken together, the observed improvements in body composition, strength, exercise capacity and BMD revealed that CM-SLCs transplantation has rejuvenating benefits in treating TD.

## Discussion

We herein report the first proof-of-concept preclinical study demonstrating that autologous SLCs can engraft, survive, increase testosterone levels and relieve the relevant symptoms of TD in an NHP model based on short-term observations. Thus, a larger cohort with long-term follow-up needs to be performed to assess the translational feasibility of autologous SLCs transplantation for treating TD.

SLCs have been isolated and characterized from rat 29, mouse 32, pig 74 and human testes 35, 59. Unlike most rodents but consistent with human males, rhesus macaques experience significant age-related declines in circulating testosterone 75, 76, indicating that NHPs are ideal animal models for the clinical development of SLCs-based therapies. However, knowledge of NHP SLCs was lacking until the present study. Cynomolgus macaques share high genomic sequence identity with humans, and this similarity reminds us that CD271, a previously reported marker of human SLCs 35, could be used to identify and isolate SLCs from monkeys. As illustrated in the current study, CD271^+^ cells not only expressed the known SLCs markers Nestin and PDGFRα but also could continuously proliferate and differentiate into mesenchymal lineage cell types. Moreover, we demonstrated that CD271^+^ cells could differentiate into functional LCs that secreted considerable testosterone. Due to the similar characteristics between the CD271^+^ cells derived from monkey testis and human SLCs 35, this population of cells was identified as CM-SLCs. Although previous studies have demonstrated that SLCs could be isolated from testis samples of pups or young adults 29, 32, 35, one major concern needs to be addressed regarding whether SLCs can be isolated and expanded from elderly patients for autologous transplantation because TD is an age-associated disease 61. In the present study, we successfully isolated CM-SLCs using minimal testicular biopsies from elderly cynomolgus monkeys and produced these cells at a clinical scale (more than 3×10^11^ cells at P10) in the expansion medium, which provides new clues for further development of human SLCs transplantation as a clinical therapy for TD.

Many studies have shown that SLCs transplantation significantly restored testosterone production and improved spermatogenesis in animal models, including aged or LCs-disrupted rodents 31-34. Because large-animal models are critical for examining the safety and feasibility of experimental therapies before they are translated to the clinic 41, we screened 30 elderly monkeys (19 to 23 years old) and enrolled 11 of them with the indicators of age-associated TD for evaluating the therapeutic potential of autologous cell transplantation. CM-SLCs, rather than CM-DFs, significantly increased the serum testosterone level, indicating that these cells could potentially be used to treat aging-related TD. The circulating concentration of testosterone exhibits the appropriate circadian rhythm 76 and can be modulated by the HPG axis 3. Here, we observed that monkeys transplanted with CM-SLCs appeared to display a normal daily rhythm of testosterone secretion. Besides, when the monkeys in the SLCs-transplanted group were administered decapeptyl, secretion of testosterone showed the typical pattern of increasing at the beginning and decline thereafter, suggesting that transplanted CM-SLCs were under the regulation of the HPG axis. Although testosterone replacement can help relieve the symptoms of hypogonadism 72, 73, this treatment can result in a reduction in intratesticular testosterone production and disruption of spermatogenesis 18-20. In the present study, we demonstrated that CM-SLCs transplantation partially recovered spermatogenesis in TD monkeys. Although there was no statistical significance, semen parameters in monkeys transplanted with CM-DFs showed the growth trend, compared with the pre-transplanted animals. We speculate this might be due to the paracrine benefits of CM-DFs to the ageing microenvironments. As previously reported, DFs could secret a large number of cytokines, including fibroblast growth factor 2 (FGF2), stem cell factor (SCF) and hepatocyte growth factor (HGF) 77. FGF2 is bona fide self-renewal factors for SSCs. Moreover, FGF2 also modifies germline niche functions right for spermatogonial differentiation 78. It has been reported that SCF is required for maintenance of differentiated germ cells 79. In addition, HGF could modulate germ cell metabolic activities *in vitro*, promoting germ cell proliferation while inhibiting apoptosis of germ cells 80. Collectively, these studies indicated that the transplanted CM-DFs may promote spermatogenesis through paracrine factors to some degree. Moreover, for the first time, the short-term restoration of testosterone levels after CM-SLCs transplantation ameliorated the TD-relevant symptoms with respect to body composition, strength, exercise capacity and BMD. These results indicated that SLCs have the potential to alleviate relevant symptoms of TD, which have not yet been examined for SLCs in previous studies 31-34. Along with testosterone recovery, we also observed that monkeys transplanted with CM-SLCs showed an improving tendency in sexual function (Figure S9D-K). While these data were not statistically significant, they exhibited a high degree of animal-to-animal consistency and thus would be likely to reach statistical significance in studies involving larger numbers of animals in the future 43.

Chemotherapy and radiation treatments for cancer or other conditions can result in the loss of both spermatogenesis and LC function 81. Therefore, most patients suffer from TD, increased body fat, decreased libido and infertility 82. Currently, adult patients have the option to preserve their future fertility by semen cryopreservation, while for prepubertal boys faced with gonadotoxic treatments, testicular tissue fragments can be cryopreserved for potential autografting, *in vitro* maturation, and SSCs auto-transplantation with the aim of fertility restoration 83-85. However, only a few attentions have been paid to the dysfunction of LCs 86. From the current data, cryopreservation of a testicular biopsy taken before the initiation of cancer treatment followed by *in vitro* propagation of SLCs and subsequent auto-transplantation of these stem cells after cancer treatment, could be suggested as an approach to treat TD-associated symptoms in cancer survivors. In addition, the risk of malignant cell contamination and disease re-introduction to the host should be noticed in the autologous SLCs transplantation scheme, especially for patients with metastasizing tumors. In the present study, flow cytometry sorting was conducted based on the difference in cell surface markers to isolate and enrich CD271^+^ SLCs, which may provide a method to remove malignant cells.

The principal limitation of this study is the recovery of testosterone levels in a relatively short period after CM-SLCs transplantation (8 weeks), which may be due to the dramatic decrease in the number of RFP^+^ cells in the interstitium 12 weeks after transplantation (Figure S6). Previous studies have demonstrated that stem cells derived from aged animals showed cell-autonomous defects in proliferation and differentiation compared with their counterparts from young donors 87, 88. Moreover, the aging microenvironment has been shown to dictate stem cell behavior 89. Similarly, Curley M, et al. reported that age-related disruption of the testicular microenvironment or wider endocrine milieu likely plays a critical role in LC dysfunction 90. To improve the therapeutic effects of SLCs transplantation, we need to develop better ways to rejuvenate aged SLCs *in vitro* and improve the aging microenvironment *in vivo* in future studies.

## Conclusion

We first isolate and characterize cynomolgus monkey SLCs and demonstrate that autologous SLCs transplantation can increase testosterone levels and relieve relevant symptoms of TD in a primate model for at least 8 weeks. These findings support the need for large-scale studies with long-term follow-up to assess the translational feasibility of autologous SLCs for TD patients.

## Supplementary Material

Supplementary figures, tables, movie legend.

Supplementary movie.

## Abbreviations

17β-HSD: 17-beta-hydroxysteroid dehydrogenase
3β-HSD: 3-beta-hydroxysteroid dehydrogenase
bFGF: basic fibroblast growth factor
BMD: bone mineral density
BSA: bovine serum albumin
BT: bioavailable testosterone
CM-DFs: cynomolgus monkey dermal fibroblasts
CM-SLCs: cynomolgus monkey SLCs
CYP11A1: cytochrome p450 family 11 subfamily A member 1
DXA: dual-energy X-ray absorptiometry
EGF: epidermal growth factor
FBS: fetal bovine serum
FT: free testosterone
GnRHa: agonist of gonadotropin-releasing hormone
IGF1: insulin-like growth factor 1
ITS: insulin-transferrin-sodium selenite
LCs: Leydig cells
LH: luteinizing hormone
LHR: luteinizing hormone receptor
LIF: leukemia inhibitory factor
NEAA: nonessential amino acids
NHP: non-human primate
OSM: oncostatin M
PBS: phosphate-buffered saline
PDGF-AA: platelet-derived growth factor AA
PDGF-BB: platelet-derived growth factor BB
RFP: red fluorescent protein
SF-1: steroidogenic factor 1
SLCs: stem Leydig cells
SSCs: spermatogonial stem cells
StAR: steroidogenic acute regulatory protein
TD: testosterone deficiency
TRT: testosterone replacement therapy
TT: total testosterone
