# Supplementary material for: Restorative functions of Autologous Stem Leydig Cell transplantation in a Testosterone-deficient non-human primate model
Source: Theranostics. 2020 Jul 9;10(19):8705–20. doi: 10.7150/thno.46854 (PMC7392013; doi:10.7150/thno.46854)
Supplement: Supplementary file 1 — Supplementary figures, tables, movie legend. [file thnov10p8705s1.pdf]

Supplementary Materials:

Figure S1. CM-SLCs diminish with increasing age.

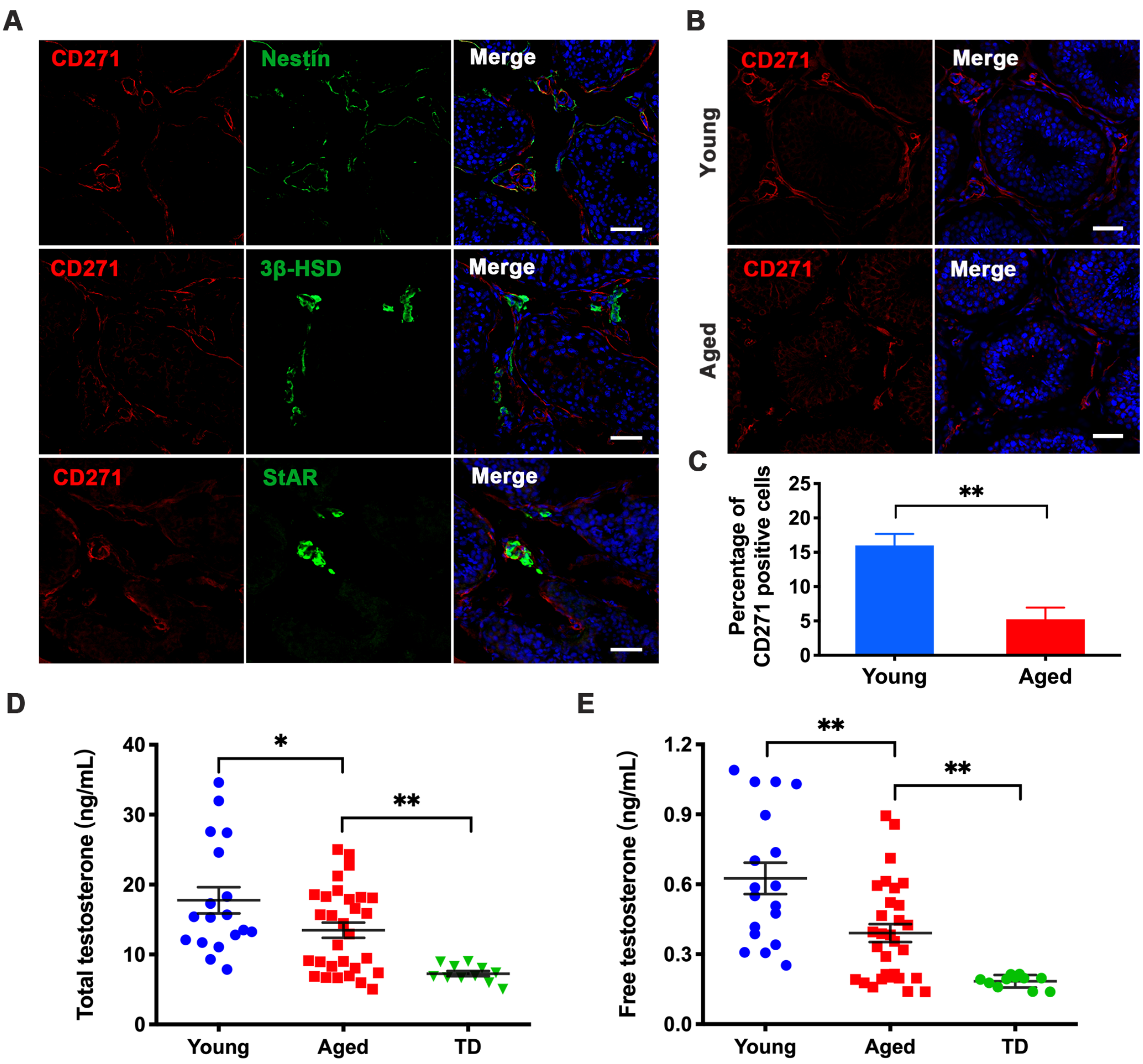

(A) Immunohistochemical analyses showed that CD271 was co-expressed with Nestin but was not with 3β-HSD or StAR in monkey testes. Scale bar, 50 μm. (B) Testis sections from young (8 to 10 years old) and aged (19 to 23 years old) male cynomolgus monkeys were immunoassayed for CD271. Images represent the results obtained from three independent experiments. Scale bars, 50 μm. (C) Quantification of $CD271^+$ cells in testis sections from the young and aged groups. Three sections per slide and three slides per testis were counted. Scale bars, 50 μm. (D, E) Total testosterone and free testosterone levels were analyzed in the

young (n=18), aged (n=30), and TD (n=11) groups. Data are expressed as the mean ± sem and were analyzed by Student's *t*-test. *P < 0.05, **P < 0.01.

Figure S2. The differentiation ratio of CM-SLCs towards LCs.

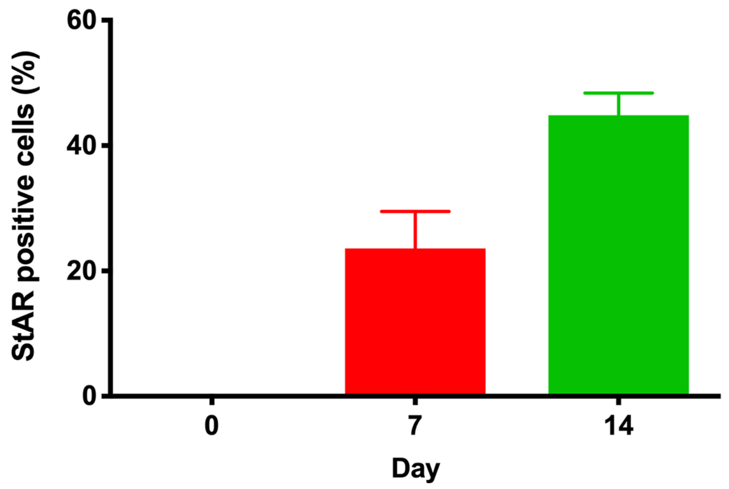

The proportion of differentiated cells was analyzed at indicated time points, as determined by immunofluorescence staining of LCs marker StAR (n=3). Data are expressed as the mean ± sem.

Figure S3. Long-term expansion, tumorigenicity assessment and chromosomal analysis of CM-SLCs.

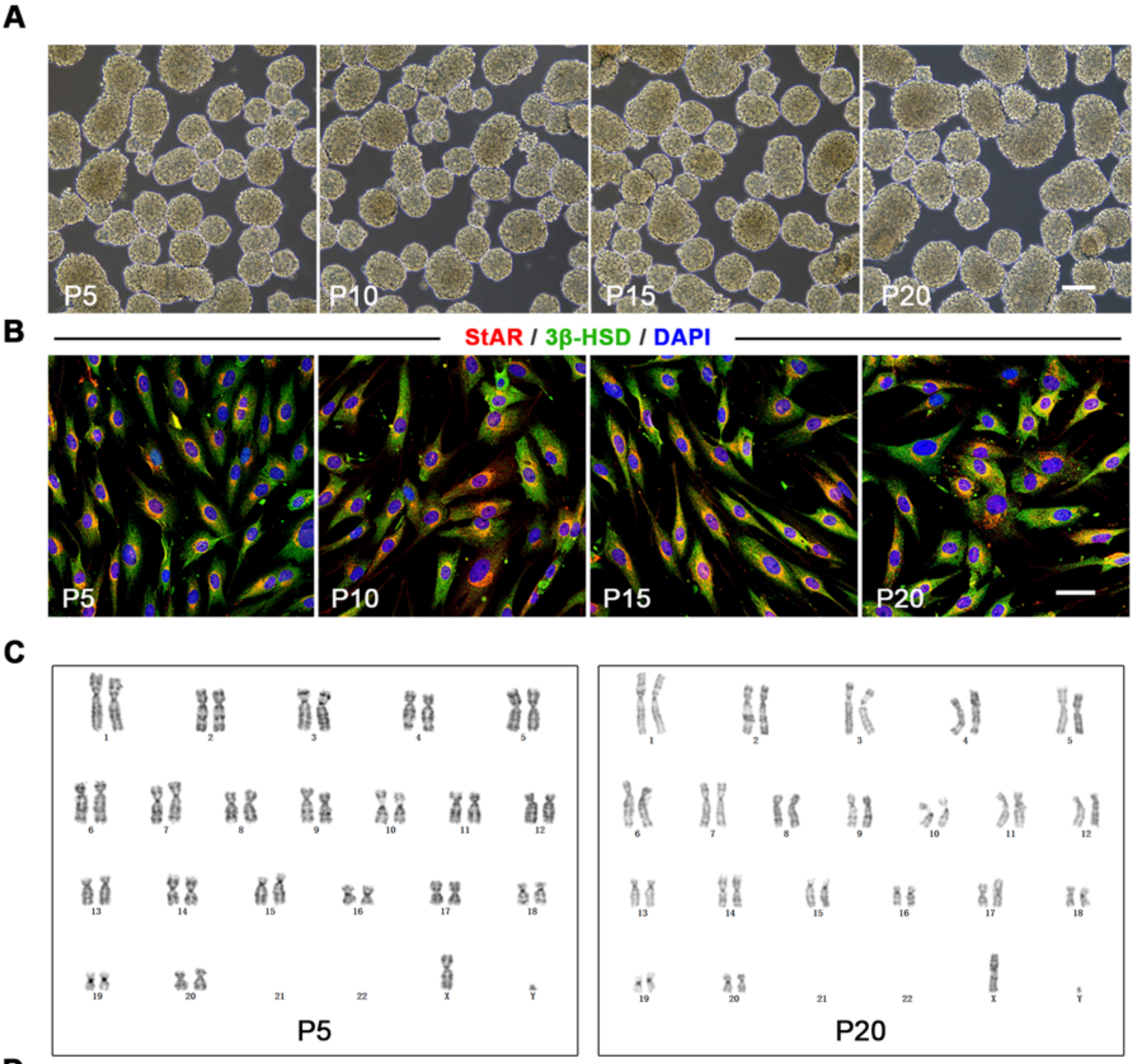

D

	10^4	10^5	10^6	10^7
MA-10	3/3	3/3	3/3	-
CM-SLCs, P5	0/3	0/3	0/3	0/3
CM-SLCs, P20	0/3	0/3	0/3	0/3

(A) Phase-contrast micrographs of CM-SLCs at various passages (P5, P10, P15, and P20). Scale bar, 100 μm. (B) Expanded CM-SLCs at different passages (P5, P10, P15, and P20)

were differentiated for 14 days. The cells were stained for StAR and 3β-HSD. Scale bar, 50 μm. (C) Karyotypic stability of expanded CM-SLCs at passage 5 (P5) and passage 20 (P20) was assessed. (D) Expanded CM-SLCs and mouse Leydig tumor MA-10 cells were transplanted subcutaneously into immunodeficient NCG mice. Tumor formation was scored upon detection or (when no tumor had been detected) at 3 months, and the number of mice harboring tumors *vs.* the total number of mice was scored.

Figure S4. Multilineage differentiation of CM-SLCs.

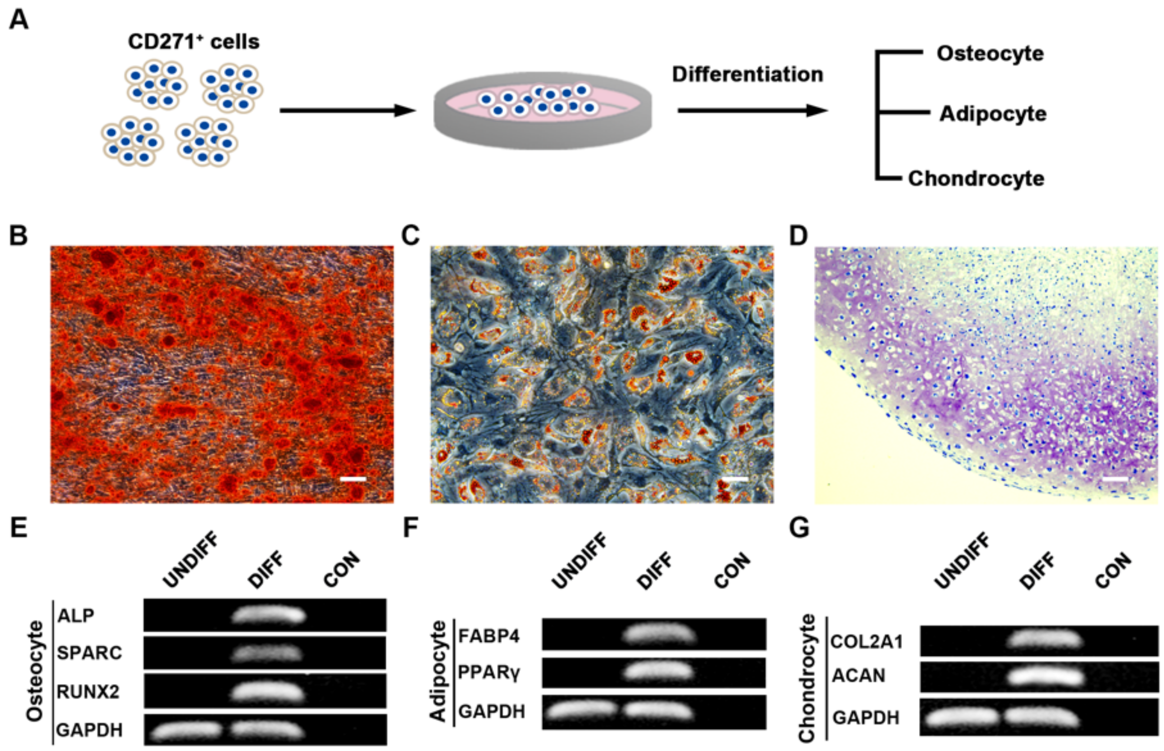

(A) Schematic of the experimental procedure used to induce multilineage differentiation. (B-D) Representative micrographs of histological staining showing osteocytes (Alizarin Red) (B), adipocytes (Oil Red O), (C) and chondrocytes (Toluidine Blue) (D). Scale bar, 50 μm (B, C), 100 μm (D). (E-G) RT-PCR analysis of osteogenic (ALP, SPARC and RUNX2) (E), adipogenic (FABP4 and PPARγ) (F) and chondrogenic (COL2A1 and ACAN) (G) markers in undifferentiated CM-SLCs (UNDIFF) and differentiated CM-SLCs (DIFF).

Figure S5. CM-SLCs transplantation decreases LH levels in monkeys with TD.

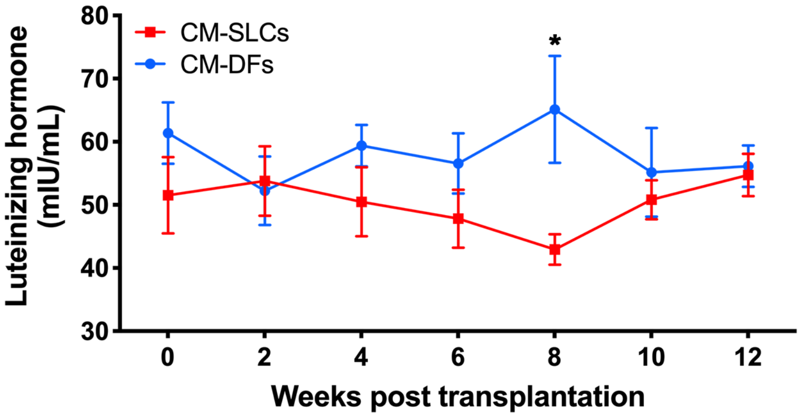

LH was measured at the indicated time points after CM-SLCs transplantation. Data are expressed as the mean ± sem and were analyzed by Student's *t*-test; * $P < 0.05$.

Figure S6. Description of the transplanted CM-SLCs in the testes of recipient monkeys.

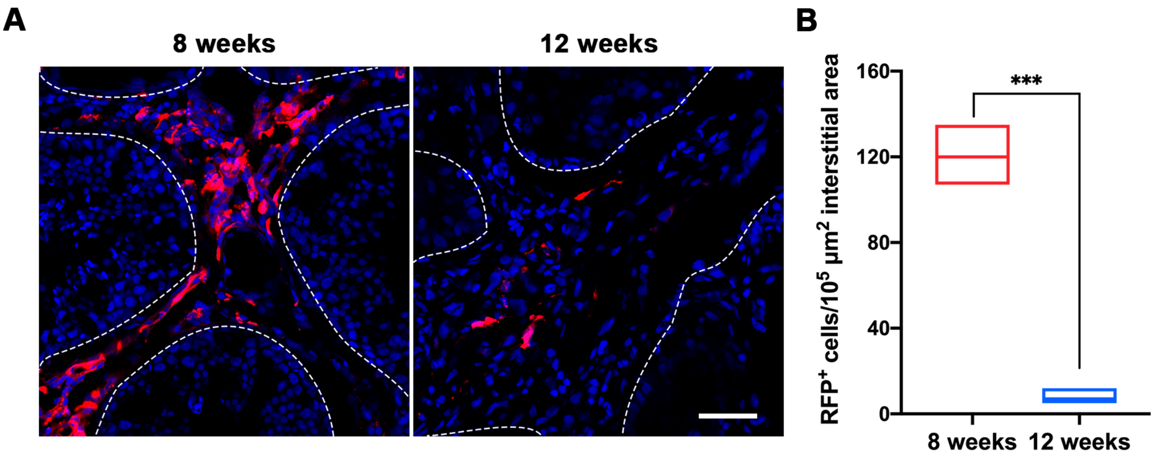

(A) Testis sections were analyzed 8 and 12 weeks after CM-SLCs transplantation. Scale bars, 50 μm.

(B) Quantification of RFP^+ CM-SLCs in testis sections. Three sections per slide and three slides per testis were counted. Data are expressed as the mean ± sem and were analyzed by Student's *t*-test. ***$P < 0.001$.

Figure S7. Marker expression in RFP-positive cells.

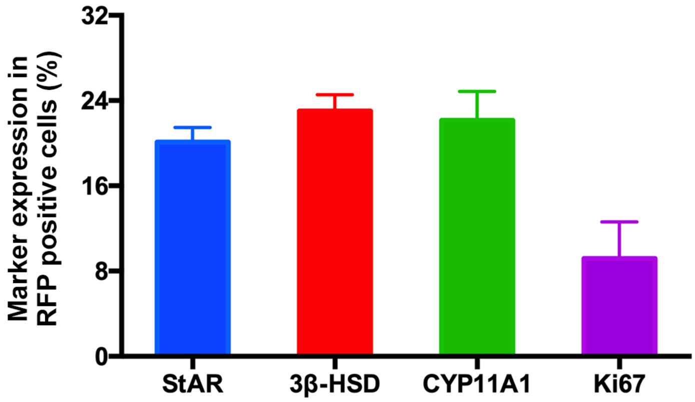

The percentages of StAR⁺, 3β-HSD⁺, CYP11A1⁺, and Ki67⁺ cells from transplanted RFP⁺ CM-SLCs were analyzed. Data are expressed as the mean ± sem.

Figure S8. Vascularization of transplanted CM-SLCs by the host vascular system.

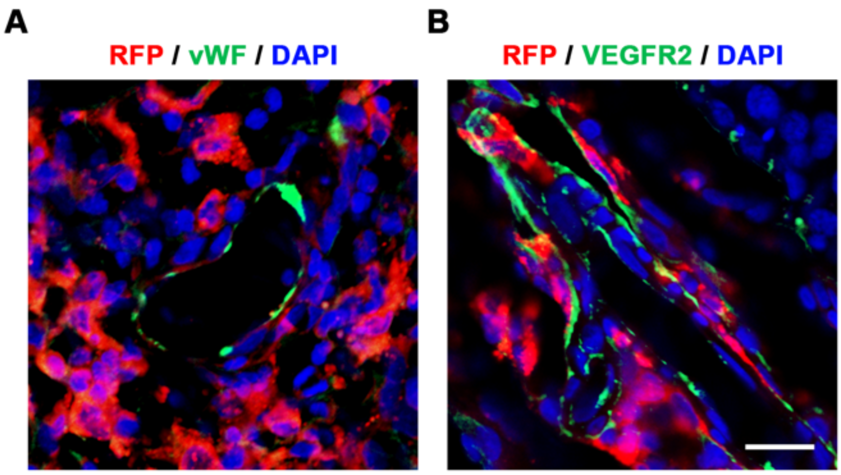

(A-B) Expressions of the vascular endothelial markers vWF (A) and VEGFR2 (B) in the transplanted testis. Scale bar, 25 μm.

Figure S9. Effects of CM-SLCs transplantation on body composition and sexual behavior parameters.

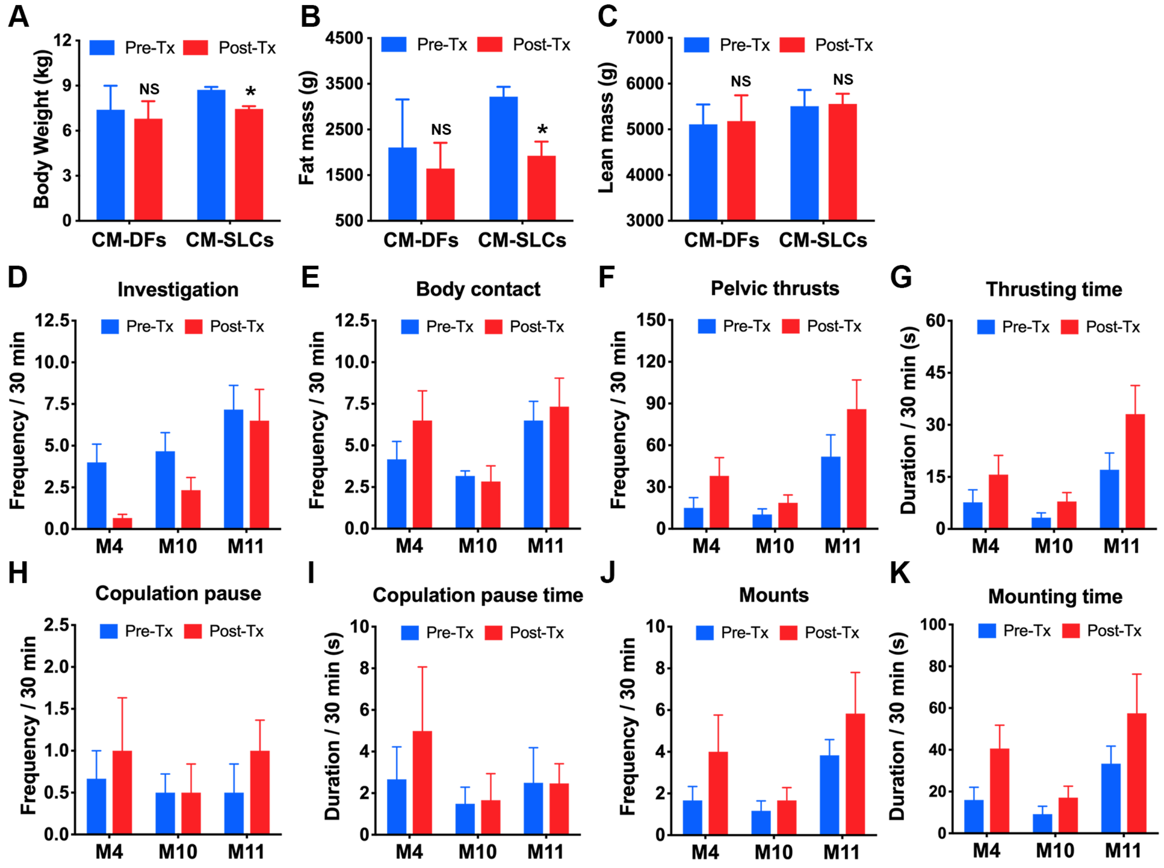

(A-C) The body weight (A), fat mass (B) and lean mass (C) of the studied monkeys were measured. (D-K) Investigation (D), body contact (E), pelvic thrusts (F), pelvic thrusting time (G), copulation pause (H), copulation pause time (I), mounts (J), and mounting time (K) of monkeys M4, M10, and M11 were compared before and after cell transplantation. Pre-Tx=before transplantation, Post-Tx=after transplantation. Data are expressed as the mean ± sem and were analyzed by Student's *t*-test. *P < 0.05. NS=not significant.

Table S1. Summary of all experimental animals, treatments and results.

**Animal Number**		**Birth Date**	**Age (year)**	**Body Weight (kg)**	**Biopsy Weight (g)**	**Transplanted Cell Type**	**Cell Volume (μL/side)**	**Number of Total Cells ($\times 10^6$/side)**
M1	392G	1999/5/16	20	10.6	0.55	CM-DFs	500	14.8
M2	429G	1999/9/11	20	6.7	0.48	CM-DFs	500	13.9
M3	431G	1996/6/15	23	5.8	0.61	CM-DFs	500	19.8
M4	433G	1998/2/10	21	5.8	0.56	CM-DFs	500	21.3
**mean**	-	-	21	7.23	0.55	-	500	17.45
**sem**	-	-	0.71	1.15	0.03	-	0	1.83
M5	387G	1997/4/15	22	8	0.54	CM-SLCs	500	17.2
M6	389G	1996/5/5	23	6.8	0.45	CM-SLCs	500	11.5
M7	390G	1997/7/11	22	8.3	0.52	CM-SLCs	500	18.8
M8	391G	1997/6/18	22	7.1	0.55	CM-SLCs	500	14.6
M9	393G	1997/3/6	22	9	0.49	CM-SLCs	500	19.2
M10	430G	1997/6/25	22	9	0.63	CM-SLCs	500	19.4
M11	432G	1999/3/21	20	8.7	0.46	CM-SLCs	500	20.5
**mean**	-	-	21.86	8.13	0.52	-	500	17.31
**sem**	-	-	0.34	0.34	0.02	-	0	1.21

Table S2. Safety evaluation of testis biopsy.

	0 w	2 w	4 w	n	Unit
**TT**	5.612 ± 0.8089	4.634 ± 0.833	6.172 ± 0.653	11	ng/mL
**Hs-CRP**	1.661 ± 0.363	2.722 ± 0.981	1.481 ± 0.254	11	mg/L
**White blood cell**	10.804 ± 0.855	9.179 ± 0.883	9.435 ± 0.686	11	×10^9/L
**Neutrophil**	6.23 ± 0.749	* 4.231 ± 0.388	4.936 ± 0.451	11	×10^9/L
**Lymphocyte**	3.504 ± 0.392	3.775 ± 0.548	3.562 ± 0.505	11	×10^9/L
**Monocyte**	0.745 ± 0.063	0.685 ± 0.087	0.58 ± 0.065	11	×10^9/L
**Eosinophil**	0.313 ± 0.104	0.491 ± 0.112	0.337 ± 0.062	11	×10^9/L
**Basophil**	0.02 ± 0.009	0.015 ± 0.003	0.019 ± 0.007	11	×10^9/L
**Red blood cell**	5.705 ± 0.19	5.423 ± 0.138	5.7 ± 0.163	11	×10^12/L
**Hemoglobin**	136.545 ± 4.15	128.909 ± 3.558	134.545 ± 3.407	11	g/L
**Platelet**	378.273 ± 30.752	372.636 ± 26.29	* 436.273 ± 28.736	11	×10^9/L

TT: total testosterone; Hs-CRP: high-sensitivity C-reactive protein. Data are expressed as the mean ± sem, and were analyzed by one-way ANOVA; * $P < 0.05$.

Table S3. Safety evaluation of CM-SLCs transplantation.

	0 w	4 w	8 w	n	Unit
**White blood cell**	8.476 ± 1.068	11.236 ± 2.095	10.709 ± 1.879	7	×10^9/L
**Neutrophil**	3.67 ± 0.574	5.739 ± 1.405	6.226 ± 1.882	7	×10^9/L
**Lymphocyte**	3.816 ± 0.763	4.386 ± 0.852	3.367 ± 0.542	7	×10^9/L
**Monocyte**	0.596 ± 0.096	0.771 ± 0.111	0.757 ± 0.163	7	×10^9/L
**Eosinophil**	0.381 ± 0.096	0.323 ± 0.076	0.349 ± 0.155	7	×10^9/L
**Basophil**	0.027 ± 0.01	0.017 ± 0.005	0.011 ± 0.003	7	×10^9/L
**Red blood cell**	5.527 ± 0.227	5.699 ± 0.207	6.134 ± 0.259	7	×10^12/L
**Hemoglobin**	130.857 ± 3.894	133.429 ± 4.14	144.286 ± 5.051	7	g/L
**Platelet**	401.714 ± 43.499	425.143 ± 52.597	323.286 ± 27.121	7	×10^9/L
**ALT**	28.929 ± 8.733	16.8857 ± 5.266	28.457 ± 10.397	7	IU/L
**AST**	42.214 ± 5.61	37.2 ± 3.368	32.914 ± 3.601	7	IU/L
**Alkaline phosphatase**	103.286 ± 5.272	109.286 ± 6.893	112.286 ± 8.199	7	IU/L
**Cholinesterase**	7205.143 ± 1223.578	7285.714 ± 1004.831	7610.143 ± 1287.522	7	IU/L
**LDH**	687.857 ± 91.882	* 342 ± 71.321	564 ± 101.935	7	IU/L
**γ-glutamyl transferase**	64.757 ± 14.852	62.1 ± 14.92	69.729 ± 15.22	7	IU/L
**Total protein**	67.457 ± 5.299	65.657 ± 6.252	61.886 ± 6.821	7	g/L
**Albumin**	27.129 ± 2.71	28.5 ± 1.799	27.171 ± 4.101	7	g/L
**Globulin**	55.029 ± 2.823	52.543 ± 3.578	49.5 ± 3.218	7	g/L
**Prealbumin**	238.714 ± 19.823	204.857 ± 24.131	208.714 ± 24.52	7	mg/L

**Total bilirubin**	4.286 ± 0.627	3.943 ± 0.426	3.043 ± 0.361	7	μM
**Direct bilirubin**	0.686 ± 0.108	0.771 ± 0.144	0.857 ± 0.221	7	μM
**Indirect bilirubin**	3.6 ± 0.582	3.171 ± 0.392	2.743 ± 0.153	7	μM
**Total bile acid**	6.086 ± 2.478	2.657 ± 0.947	6.814 ± 3.077	7	μM
**Creatinine**	81.643 ± 6.589	75.943 ± 6.267	75.3 ± 8.448	7	μM
**Urea**	5.786 ± 0.402	5.486 ± 0.493	6.771 ± 0.861	7	nM
**Uric acid**	4.714 ± 0.606	5.143 ± 0.553	3.429 ± 0.429	7	μM
**Creatine kinase**	215.143 ± 110.955	96.429 ± 13.535	78.857 ± 10.631	7	IU/L
**Creatine kinase isoenzyme**	73.286 ± 16.586	66.429 ± 9.429	74.143 ± 8.849	7	IU/L
**HBDH**	306.857 ± 32.349	185.571 ± 29.258	264 ± 37.925	7	IU/L
**AFP**	0.059 ± 0.018	-	0.063 ± 0.03	7	μg/L
**HCG**	0.104 ± 0.068	-	0.039 ± 0.039	7	mIU/mL
**PSA**	0.327 ± 0.068	-	0.431 ± 0.095	7	ng/mL

ALT: alanine aminotransferase; AST: aspartate aminotransferase; LDH: lactate dehydrogenase; HBDH: hydroxybutyrate dehydrogenase; AFP: alpha-fetoprotein; HCG: β-human chorionic gonadotropin; PSA: prostatic specific antigen. Data are expressed as the mean ± sem, and were analyzed by one-way ANOVA or Student's *t*-test. * P < 0.05.

Table S4. Primers used to amplify the transcripts for RT-PCR analysis.

Gene	Forward Primer	Reverse Primer
LHR	CCTGACAAGTCGTTACAA	TATGAGCAGCAGATAGAGT
3β-HSD	AGGACAGTTCTATTACATCTC	TACATCAGGGCTAAAGGA
StAR	GGAGTGGAACCCTAATGT	ATCTCGTGAGTAATGAATGTATC
CYP11A1	ATCAATATGCTGGAGAACTT	CAGGATGAGGTTGAATGT
17β-HSD	AGGAGTGTGAAGATTATAC	TGATGTTACAATGGATGA
SF-1	AATGCCTACAGTTATTCC	GAGTCAGGTCTTCTATCT
COL2A1	AGCAAGAGCAAGGAGAAGA	GGAGCCAGGTTGTCATCT
ACAN	TACGATGTCTACTGCTATGT	GAAGGTGAACTGCTCAAG
PPARγ	CCATTCACAAGAACAGAT	CAGAATAATAAGGTGGAGAA
FABP4	GATCATCAGTGTGAATGG	TAAGGTTATGGTGCTCTT
ALP	CATAACATCAGGGACATTG	ATCTCATACTCCACATCAG
SPARC	AGAGGGATGAAGACAACA	GCTTCTCATTCTCATGGAT
RUNX2	ACCATAACCGTCTTCACAA	GAGGTCCATCTACTGTAACTT
GAPDH	CTCTGGTAAAGTGGATATTG	GGTGGAATCATACTGGAA

Table S5: Primary and secondary antibodies used for immunostaining or flow cytometry analysis.

Antibodies	Dilution	Distributor (Cat. NO)
Mouse anti-3β-HSD	1:100	Santa Cruz (sc515120)
rabbit anti-LHR	1:150	Santa Cruz (sc25828)
rabbit anti-SF-1	1:150	Abcam (ab65815)
Rabbit anti-StAR	1:100	GeneTex (GTX105716)
Mouse anti-StAR	1:50	Santa Cruz (sc166821)
Rabbit anti-17β-HSD	1:150	GeneTex (GTX114480)
Rabbit anti-CYP11A1	1:200	GeneTex (GTX56293)
Goat anti-CYP11A1	1:50	Santa Cruz (sc18043)
Rabbit anti-CD271	1:100	Promega (G3231)
Rabbit anti-Nestin	1:100	Millipore (ABD69)
Mouse anti-Nestin	1:100	Arigo (arg52345)
Rabbit anti-PDGFRα	1:200	Abcam (ab203491)
Mouse anti-CD31	1:100	Santa cruz (sc-13537)
Rabbit anti-Cx43	1:500	CST (3512)
Rabbit anti-SCP1	1:200	Abcam (ab15090)
Rabbit anti-SCP3	1:200	Abcam (ab15093)
Rabbit anti-vWF	1:200	GeneTex (GTX26994)
Rabbit anti-VEGFR2	1:200	CST (2479S)
Goat anti-rabbit IgG AF488	1:1000	Invitrogen (A11034)
Goat anti-rabbit IgG AF594	1:1000	Invitrogen (A11037)
Goat anti-rabbit IgG AF647	1:1000	Invitrogen (A21244)
Goat anti-mouse IgG AF488	1:1000	Invitrogen (A11001)

Goat anti-mouse IgG AF594	1:1000	Invitrogen (A11032)
Donkey anti-goat IgG AF488	1:1000	Invitrogen (A11055)
Mouse anti-CD271 AF647	1:50	BD (560877)

Movie S1. CM-SLCs transplantation by ultrasound-guided testis injection.

Ultrasound-guided testis injection for transplantation of CM-SLCs or CM-DFs. Using a 13-MHz linear superficial ultrasound probe on a Logiq E9 ultrasound instrument, we guided a 25G needle into the testicular interstitium and infused a suspension of CM-DFs or CM-SLCs into recipient cynomolgus monkey testes. This movie shows the guidance and positioning of the echo-dense (white) injection needle into the testis space and subsequent injection of the donor cell suspension using positive pressure. The donor cell suspension contained a microbubble ultrasound contrast agent, which enabled real-time visual monitoring of the injection progress (yellow). This movie shows the injected cell suspension radiating from the needle tip and subsequently spreading into the testicular interstitium.
